# 2-Deoxy-D-Glucose and ES-936 sensitize cancer- but not normal cells to both low- and high LET irradiation

**DOI:** 10.3389/fonc.2025.1633299

**Published:** 2025-08-18

**Authors:** Katja Kratz, Henrieke Förster, Kira Vogel, Marco Durante, Burkhard Jakob

**Affiliations:** ^1^ Department of Biophysics, GSI Helmholtzzentrum für Schwerionenforschung, Darmstadt, Germany; ^2^ Department of Biology, Technical University of Darmstadt, Darmstadt, Germany; ^3^ Department of Condensed Matter Physics, Technical University of Darmstadt, Darmstadt, Germany

**Keywords:** radiotherapy, high-LET, cancer, metabolism, DNA-repair, carbon-ions, glycolysis, 2-deoxy-D-glucose

## Abstract

**Introduction:**

Metabolic differences of normal- and cancer cells represent an important target for the development of novel cancer treatment strategies. Given that radiotherapy constitutes one of the primary treatment modalities for solid cancers, the targeting of cancer cell metabolism to enhance their sensitivity to irradiation emerges as a promising approach. The utilization of glycolysis even under aerobic conditions in cancer cells presents a unique target to deprive cancer cells of energy and metabolites required not only for their rapid cell growth but also for the repair of irradiation induced DNA damage. Furthermore, cancer cells have been observed to exhibit elevated levels of reactive oxygen species and potentially react more sensitively to an induced disturbance of the redox balance, especially after irradiation mediated oxidative stress. Overall, interference with aerobic glycolysis and the oxidative stress response could potentiate the anti-proliferative and cytotoxic effects of cancer cell irradiation, while sparing normal cells.

**Methods:**

To analyze the effect of inhibitors targeting the cellular metabolism and redox balance, normal fibroblast- and cancer cell lines were characterized using a Seahorse XFp metabolic analyzer, followed by Sulforhodamin B proliferation assays and flow cytometry based cell cycle analysis. Furthermore, NADP+/NADPH-, NAD(P)H- and ROS levels were determined using bioluminescent assays, Fluorescence Lifetime Imaging Microscopy (FLIM) and fluorescent microscopy. Radiosensitization of cell lines was assessed through clonogenic survival assays and analyses of DNA-repair efficiency via fluorescence microscopy.

**Results:**

The present study demonstrates that the glycolytic inhibitor 2-deoxy-D-glucose and the NAD(P)H:quinone oxidoreductase inhibitor ES-936 can render cancer cells more sensitive to X-rays and densely ionizing radiation (high-linear energy transfer (LET) irradiation) like alpha-particles or heavy ions but do not affect normal fibroblasts. While inhibitor-treated and low-LET (X-ray) irradiated cancer cells exhibited a decreased clonal survival, an additional DNA repair defect was observed after high-LET irradiation.

**Discussion:**

Our results imply that distinct mechanisms influence the clonal survival and DNA repair of irradiated, inhibitor-treated cancer cells in dependence of the LET. The findings of this study suggest that the combination of inhibitors targeting glycolysis and the redox balance may represent a promising strategy to enhance the sensitivity of cancer cells to both photon- and charged particle therapy.

## 1 Introduction

In mammals, a tightly regulated cellular metabolism normally fulfills all tissue specific needs. When cancer cells arise, they modulate and adapt their metabolic pathways to allow faster cell growth (1, 2). While normal cells mainly rely on oxidative phosphorylation for energy production, cancer cells prefer in many cases aerobic glycolysis for increased production of ATP and metabolites required for accelerated proliferation. Otto Warburg described this phenotype, which was termed the ´Warburg effect´ thereafter (3, 4). The discovery of the metabolic differences between normal- and cancer cells stimulated the employment of glycolytic inhibitors specifically to target cancer cells (5–7). One of these inhibitors is 2-Deoxy-D-Glucose (2-DG), a synthetic glucose analog with a hydroxyl group at C2 being replaced by a hydrogen atom. 2-DG inhibits the function of hexokinase and glucose-6-phosphate isomerase through accumulation of 2-deoxy-D-glucose-6-phosphate in the cell (8). In addition, 2-DG also affects the Pentose-Phosphate Pathway (PPP), which provides ribose 5-phosphate that serves as a source for nucleotide synthesis and NADPH for biosynthetic reactions and the intracellular redox defense, respectively (9–13). Other studies indicated that 2-DG induces cellular oxidative stress, as shown by an increased level of glutathiondisulfid (14, 15). While normal cells experience little oxidative stress, detectable through low intracellular reactive oxygen species (ROS) levels, cancer cells frequently have to handle higher levels of oxidative stress due to their increased metabolic rate (16). However, they often can modulate their metabolism to increase the production of antioxidant molecules and upregulate antioxidative enzymes to detoxify ROS (17). One enzyme with antioxidant activities is the NAD(P)H:quinone oxidoreductase 1 (NQO1), a cytosolic FAD-dependent flavoprotein that catalyzes the two electron reduction of quinones, quinines, quinoneimines and nitro aromatics using NAD(P)H as donor (18–20). In addition, NQO1 balances the NAD+/NADH ratio mainly through generation of NAD+, which is important for the activity of PARP and sirtuins that are involved in DNA repair (21). NQO1 is already present at high levels in many human solid tumors or upregulated after induction of ROS and can be irreversible inhibited by the component ES-936, thereby potentially increasing oxidative stress within cancer cells (22–24). Oxidative stress can be also induced by radiotherapy, which may impede the repair of irradiation-induced DNA damage (25). Radiotherapy is a predominant treatment modality for solid, metastasis-free cancers, with approximately 50% of patients receiving irradiation (26). In cancer radiotherapy, different qualities of irradiation can be applied. Most commonly, radiotherapy employs X-rays but these deposit most of their energy while they travel through healthy tissue before they reach the tumor. Consequently, multiple radiation fields are necessary to reach a tumor conform dose distribution at the cost of a huge volume of surrounding healthy tissue receiving lower radiation doses. In contrast, the use of charged particle irradiation inflicts an inverted depth dose profile called ´Bragg Peak´ where the released main energy hits the tumor (27). In addition, heavier charged particles as carbon ions possess a high-linear energy transfer (LET) compared to X-rays and protons, leading to enhanced biological effectiveness. The emerging dense ionization events along the tracks lead to clustered, complex DNA damage that is visible on a microscopic scale in form of DNA repair protein clusters (28–30). In general, clustered DNA damage arising from high-LET irradiation is considered a challenge and difficult for cells to repair correctly, a feature being advantageous for cancer therapy (31). Previous studies show that 2-DG alone or in combination with chemotherapeutics decreases the survival of cancer cells *in vitro* and *in vivo* (14, 32–36). Clinical studies focusing on cerebral gliomas and other solid tumors examined the combination of 2-DG with radiotherapy or chemotherapeutics (37–39). In another study, the combination of 2-DG with several inhibitors targeting the oxidative defense was used to sensitize radio-resistant cervical cancer cells (40). In contrast to previous studies, we aimed to characterize the radio-sensitizing effect of metabolic inhibitors on cancer cells after high-LET radiation and compare them with normal fibroblast cells. For this, we used the glycolytic inhibitor 2-DG to target the energy metabolism of cancer cells. In addition, we combined 2-DG with the inhibitor ES-936 to increase oxidative stress within the cells to interfere with DNA-repair processes of irradiation induced DNA damage. We show that cancer cells react more sensitive to low- and high-LET irradiation than normal cells when treated with a combination of the glycolytic inhibitor 2-DG and the NQO1 inhibitor ES-936. Our results demonstrate that, in comparison to normal cells, inhibitor-treated cancer cells exhibited a reduced clonogenic survival following low- and high-LET irradiation. Furthermore, we observed that inhibitor-treated cancer cells that had been subjected to high-LET irradiation exhibited an additional DNA repair defect 24h post irradiation. These results suggest that there are distinct inhibitor-induced mechanisms that function in parallel in cancer cells, impacting clonogenic survival and DNA repair in response to low- and high-LET irradiation, respectively. Consequently, a simultaneous targeting of glycolysis and oxidative defense could provide an opportunity to improve radiotherapy, including a treatment with carbon-ions.

## 2 Results

### 2.1 Normal fibroblast- and cancer cell lines do not exhibit a strong metabolic preference for ATP production

**Figure 1:**
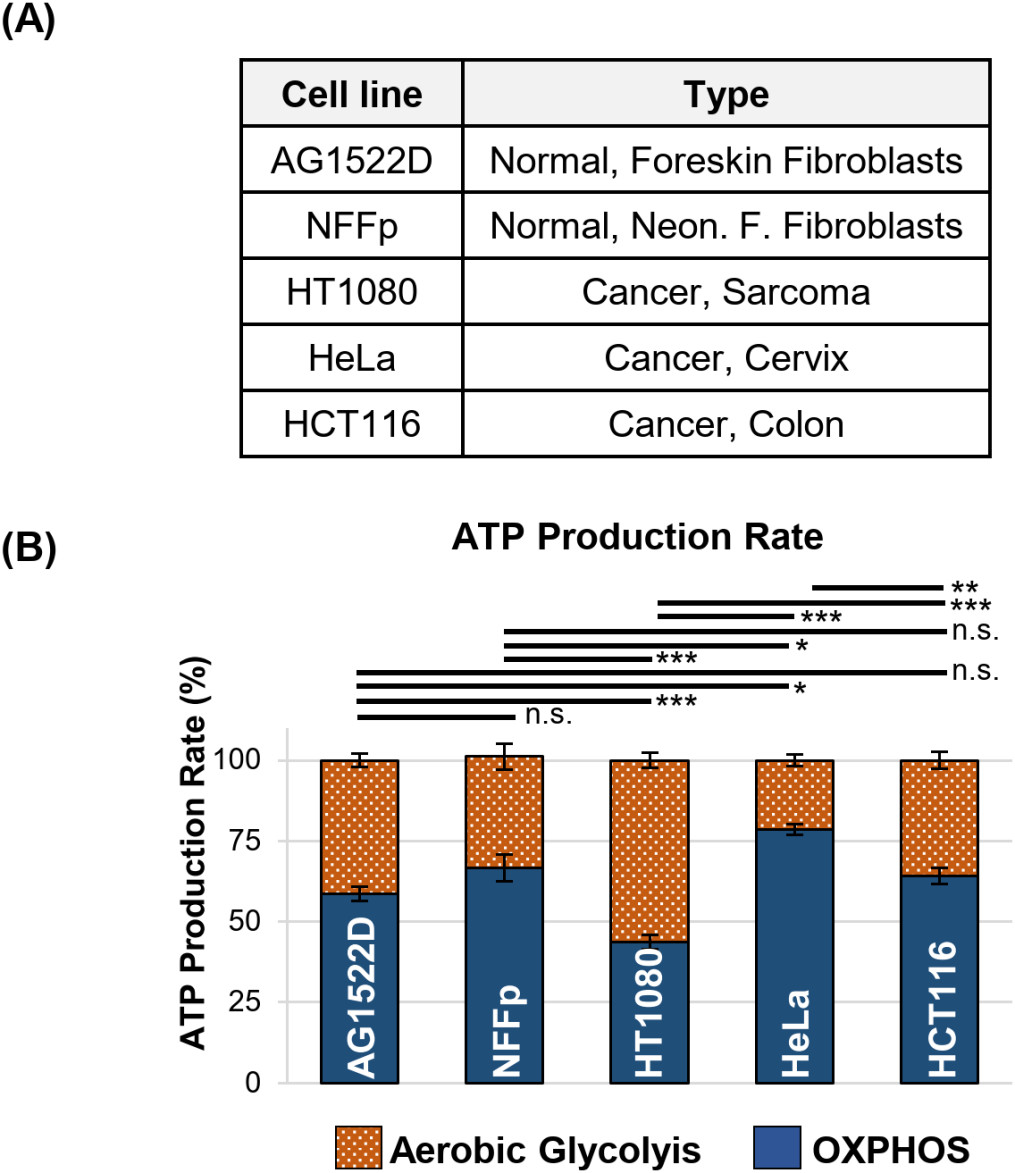
Normal fibroblast- and cancer cell lines do not exhibit a strong metabolic preference for ATP production in cell culture. **(A)** Human fibroblast- and cancer cell lines used for testing their metabolic preferences. **(B)** Relative ATP production rate originating from oxidative phosphorylation (OXPHOS) and aerobic glycolysis of the indicated cell lines measured in a Seahorse XFp metabolic analyzer with a Real-Time ATP-Rate assay (N=3 for AG1522D, NFFp; N=6 for HT1080, N=4 for HeLa, HCT116; n=3 techn. replicates; error bars indicate SEM). Significance was assessed using one-way ANOVA and Tukey *post-hoc* multiple comparisons (n.s.= not significant, *p<0.05, **p<0.01; ***p<0.001).

To test whether our cancer cell lines exhibited the characteristics of the Warburg effect, we analyzed both, normal human fibroblasts AG1522D and NFF primary (NFFp) and human cancer cell lines HT1080, HeLa and HCT116 under normoxic conditions, using a Seahorse XFp metabolic analyzer and a Real-Time ATP-Rate assay to measure the total ATP production rate through oxidative phosphorylation (OXPHOS) and aerobic glycolysis (**Figure 1A**). Our results show that the tested normal fibroblast cell lines exhibited a slight preference for oxidative phosphorylation, with app. 60-65% of ATP being generated through this pathway (**Figure 1B**). In contrast, the tested cancer cell lines did not show a strict preference for aerobic glycolysis to supply ATP under standard cell culture conditions. The cervix carcinoma HeLa cells and the colon carcinoma HCT1116 cell lines gained their ATP by employing app. 20-35% of aerobic glycolysis. Only the sarcoma HT1080 cells showed a preference for this metabolic pathway, generating 55% of their ATP via aerobic glycolysis (**Figure 1B**). Significant metabolic differences between the tested cell lines were analyzed using one-way ANOVA followed by Tukey *post-hoc* multiple comparisons. While the tests showed that AG1522D and NFFp fibroblasts were not significantly different from each other, HT1080 and HeLa cell lines were significantly different to both fibroblasts cell lines (**Figure 1B**). Only the cancer cell line HCT116 did not exhibit a significant difference to the fibroblast cell lines. In addition, the cancer cell lines showed significantly different metabolisms when compared to each other, reflecting their heterogeneity. In summary, we found that both normal fibroblast cell lines and HCT116 cells exhibited a similar metabolism but HT1080 and HeLa cells were significantly metabolically different from each other and the fibroblast and HCT116 cells, respectively. Nevertheless, neither cell line exhibited a strong reliance on a single metabolic pathway for ATP production, but rather were able to employ both pathways to a certain extend.

### 2.2 Normal fibroblast- and cancer cell lines possess different metabolic spare capacities

**Figure 2:**
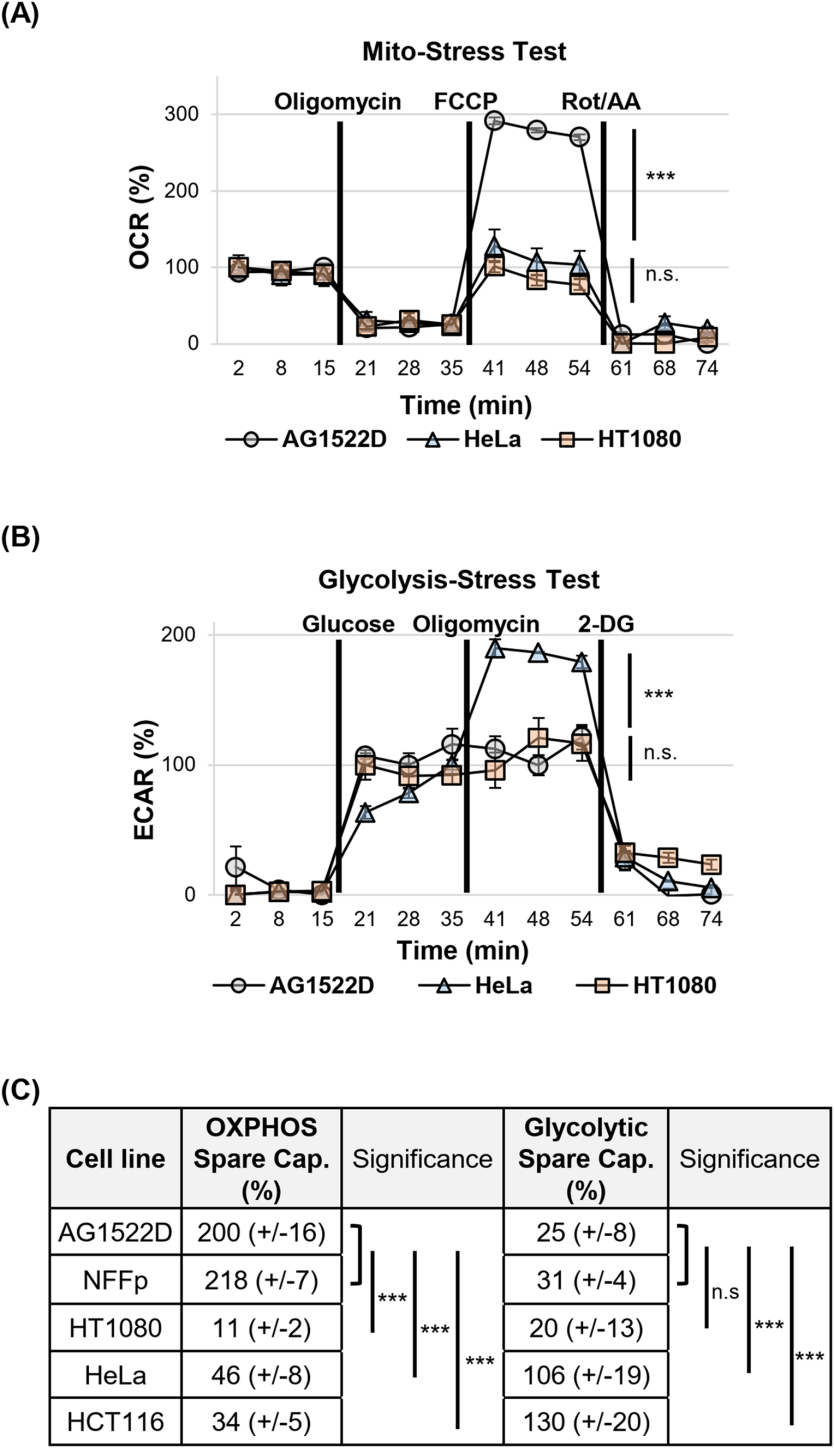
Normal fibroblast- and cancer cell lines possess different metabolic spare capacities. **(A)** Example of the oxygen consumption rate (OCR) changes during assessment of the respiratory spare capacities of normal fibroblast- and cancer cells in a Seahorse XFp metabolic analyzer using a Mito-Stress Test. **(B)** Example of the changes in the extracellular acidification rates (ECAR) during assessment of the aerobic glycolytic spare capacities of normal fibroblast- and cancer cells in a Seahorse XFp metabolic analyzer using a Glycolysis-Stress Test. **(C)** Summary of the calculated metabolic spare capacities of all cell lines tested (OXPHOS=oxidative phosphorylation. **(A–C)** OXPHOS Spare Capacity with N=2 for AGD; N=4 for NFFp; N=3 for HT1080, HeLa, HCT116; Glycolytic Spare Capacity with N=2 for NFFp, HeLa; N=3 for AG1522D, HT1080, HCT116; n=3 techn. replicates; error bars indicate SEM). Significance was assessed using one-way ANOVA and Tukey *post-hoc* multiple comparisons (n.s.= not significant, ***p<0.001).

After we noticed that both energy-producing pathways are operational in all of our cell lines, we tested them for their metabolic spare capacities. The spare capacities of a cell line reflect its maximum ability to respond to an increased energy demand or stress. We again employed the Seahorse XFp metabolic analyzer and Cell Mito Stress- and Glycolysis Stress Tests to characterize both the cellular respiratory- and glycolytic spare capacities, respectively. In **Figure 2A**, the oxygen consumption rates (OCR) of the normal fibroblast cell line AG1522D and the cancer cell lines HT1080 and HeLa during the Mito-Stress Test are shown. While normal AG1522D cells exhibited a strong FCCP-induced increase of the OCR and consequently a large respiratory spare capacity, HT1080 and HeLa cells showed only a minor increase, indicating a small respiratory spare capacity. As visible in **Figure 2B**, the extracellular acidification rate (ECAR) of HeLa cells increased significantly after addition of Oligomycin, indicating a large glycolytic spare capacity for this cell line. In contrast, AG1522D and HT1080 cells did not significantly react to the addition of Oligomycin and consequently exhibit only small glycolytic spare capacities. In summary, our tested normal fibroblast cell lines AG1522D and NFFp possess large respiratory spare capacities of app. 200-218%, while the tested cancer cells only have small respiratory spare capacities of app. 11-46% (**Figure 2C**). In contrast, the normal fibroblast AG1522D and NFFp cells exhibited only small glycolytic spare capacities of app. 25-30%, while the cancer cell lines HeLa and HCT116 had large glycolytic spare capacities of app. 106% and 130%, respectively (**Figure 2C**). The third tested cancer cell line HT1080 showed both a low respiratory- and glycolytic spare capacity with app. 11% and 20%, respectively (**Figure 2C**). Significant differences of metabolic spare capacities between the tested cell lines were analyzed using a one-way ANOVA followed by Tukey *post-hoc* multiple comparisons. Because the AG1522D and NFFp fibroblasts were not significantly different from each other, we compared their pooled data with the single cancer cell lines. Our analysis showed that all tested cancer cell lines differed significantly in their OXPHOS spare capacity from the fibroblast cells (**Figures 2A, C**). When we compared the glycolytic spare capacities of fibroblasts with the cancer cell lines, HeLa and HCT116 cancer cells differed again significantly in their spare capacity from the fibroblast cell lines (**Figures 2B, C**). Only the cancer cells HT1080 did not exhibit a significant difference of a glycolytic spare capacity when compared to fibroblasts (**Figures 2B, C**). In summary, our tested fibroblast cells exhibited large OXPHOS spare capacities and small glycolytic spare capacities while the cancer cells HeLa and HCT116 possessed small OXPHOS spare capacities and large glycolytic spare capacities. Only the cancer cell line HT1080 neither possessed an OXPHOS- nor a glycolytic spare capacity, probably because it already reached its metabolic limit to achieve its fast proliferation rate. As a result of the different metabolic spare capacities, our tested normal fibroblast- and cancer cell lines were expected to react differently to treatment with metabolic inhibitors.

### 2.3 2-DG decreases cellular proliferation, the glycolytic ATP production rate and NADP+/NADPH levels and increases ROS

**Figure 3:**
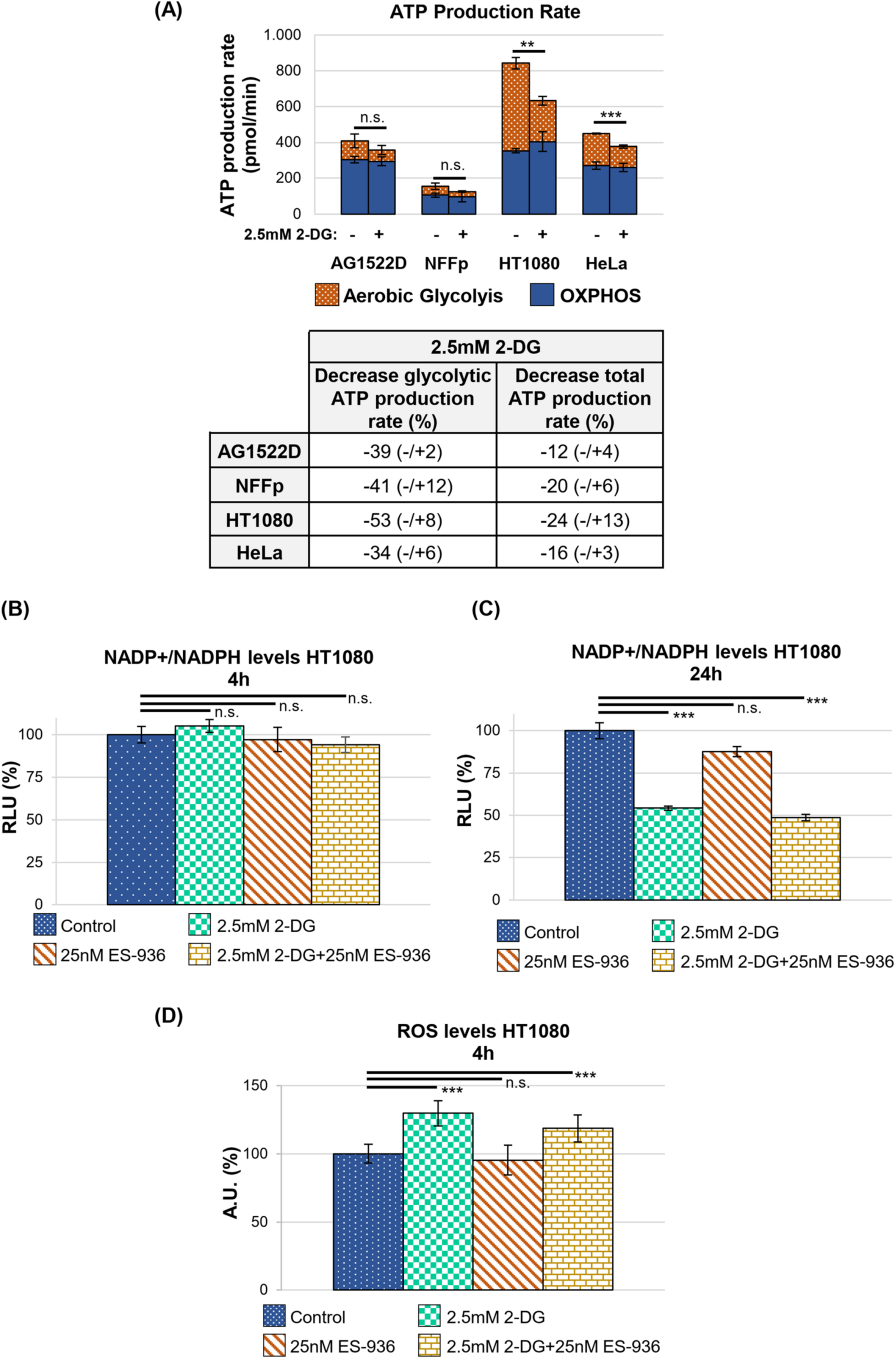
2-DG decreases the glycolytic ATP production rate as well as NADP+/NADPH levels and increases ROS production. **(A)** Upper panel: ATP production rate of indicated cell lines untreated or treated with 2.5mM 2-DG assessed by the Seahorse metabolic analyzer. Significance of changes in total ATP production rate is shown. Lower panel: 2.5mM 2-DG dependent decrease in the glycolytic and total ATP production rate, respectively, in percent (N=2 for all cell lines tested with n=2&3 techn. replicates for AG1522D, n=3 techn. replicates for NFFp, n=2&3 techn. replicates for HT1080 n=3 techn. replicates for HeLa; error bars indicate SEM). Significance was assessed using a Welch´s two-tailed t-test (n.s.= not significant, **p<0.01; ***p<0.001). **(B, C)** The total levels of NADP+/NADPH were analyzed after 4h and 24h incubation of HT1080 cells with the indicated inhibitors, respectively (each time point is N=1 with n=3 techn. replicates; error bars indicate SEM). Significance was assessed using a Welch´s two-tailed t-test (n.s.= not significant, ***p<0.001). **(D)** The levels of reactive oxygen species (ROS) were analyzed using CellRox Green after 4h incubation of HT1080 cells with the indicated inhibitors (N=2 for Control, 2.5mM 2-DG; N=1 for 25nM ES-936, 2.5mM 2-DG+25nM ES-936; analyzed images per sample: Control=22 images; 2.5mM 2-DG=30 images; 25nM ES-936 = 7 images; 2.5mM 2-DG+25nM ES-936 = 10 images; error bars indicate SEM). Significance was assessed using a Welch´s two-tailed t-test (n.s.= not significant, ***p<0.001).

Previous *in vitro* studies treated cancer cell lines with 4-20mM 2-DG (33–35), while clinical trials used 1.2-1.8mM 2-DG in combination with irradiation (37, 38). To employ inhibitor concentrations similar to those applied in clinical trials, we used 2.5mM 2-DG in culture medium containing 5mM glucose. This glucose concentration resembles the human blood glucose levels (41) and leads to an app. 1:2 ratio of 2-DG to glucose in our experiments. When our cell lines were cultured under these conditions for 24h and compared with untreated control cells, a proliferation reduction of app. 20-50% became visible (**Supplementary Figure 1A**). Using a Welch´s two-tailed t-test, the observed growth defect induced by 2.5mM 2-DG was significant for HT1080 and HCT116 cancer cells, respectively. To characterize the effect of 2.5mM 2-DG on the cellular energy metabolism, we incubated cells in either control medium or medium containing 2-DG and determined the ATP production rate. As shown in **Figure 3A**, 2-DG reduced the glycolytic ATP production rate by app. 34-53% (lower panel). HT1080 cells exhibited the strongest 2-DG dependent decrease of the glycolytic- and even total ATP production rate with 53% and 24%, respectively (**Figure 3A**, upper and lower panel). The OXPHOS-dependent ATP production rate in all cell lines tested did not significantly change after treatment with 2.5mM 2-DG (Welch´s two-tailed t-test; **Supplementary Figure 1B**). The test further revealed that the fibroblast cell line AG1522D showed no significant decrease in the glycolytic ATP production rate after treatment with 2.5mM 2-DG while NFFp cells and the tested cancer cell lines HT1080 and Hela exhibited a significant decrease in glycolytic ATP production rate. While the total ATP production rate did not significantly change for the AG1522D and NFFp fibroblast cells, it was significantly reduced for the tested HT1080 and HeLa cancer cells (**Figure 3A**, upper panel, **Supplementary Figure 1B**). In general, the OXPHOS-dependent ATP production rate did not significantly increase to compensate the loss of glycolytic ATP production, implying that cells can cope with less ATP under stress-free conditions, although all cell lines exhibited a decrease in proliferation (**Supplementary Figure 1A**). To analyze this growth delay in more detail, we determined cell cycle distribution and debris/dead cells with flow cytometry. Our with 2.5mM 2-DG treated cell lines exhibited a decreased S-phase cell population of about 34-50% and an increased G1-phase cell population of about 15-38%, suggesting that the cell lines arrest at the G1/S checkpoint (**Supplementary Figure 1C**). A Welch´s two-tailed t-test was used to analyze the significance of the observed cell cycle alterations after treatment with 2-DG. In all tested cancer cell lines, the addition of 2.5mM 2-DG induced significant changes in the cell cycle distribution, except for the G2/M phase of HCT116 cells (**Supplementary Figure 1C**). Although the amount of cell debris/dead cells increased in some of the cell lines, it represented less than 8% of the total cell population and most likely did not significantly contribute to the observed proliferation defect after 24h (**Supplementary Figure 1D**). The increase of debris/dead cells was significant for HT1080 and HCT116 cells treatment with 2.5mM 2-DG but not for HeLa cancer cells (Welch´s two-tailed t-test; **Supplementary Figure 1D**). 2-DG does not only affect glycolysis but also the linked PPP that provides ribose 5-phosphate, which serves as source for nucleotide synthesis (9, 42). Consequently, treatment of cells with 2-DG could disturb nucleotide synthesis and induce the observed proliferation defect and accumulation of cells in G1, respectively (**Supplementary Figures A, C**). In addition, the PPP also provides NADPH for biosynthetic reactions and the cellular redox defense (9, 42). To characterize the effect of 2-DG on the NADP+/NADPH level and ROS, we used the HT1080 cancer cell line that reacted most sensitive to treatment with this inhibitor. We cultured the cells in control medium or medium containing 2.5mM 2-DG for 4h and 24h, respectively, and determined their total NADP+/NADPH level using a NADP+/NADPH assay. While we could not detect any significant changes after 4h between the control and 2.5mM 2-DG treated cells using a Welch´s two-tailed t-test, we saw a significant 46% reduction of the total NADP+/NADPH level after 24h treatment with 2.5mM 2-DG (**Figures 3B, C**). To complement this experiment, we analyzed ROS in 2-DG treated HT1080 cells because the observed decrease of NADP+/NADPH levels (**Figure 3C**) can negatively affect the cellular redox defense, leading to an increase of oxidative stress. To analyze ROS within the cells, the fluorescent probe CellRox Green was added after a 4h treatment with 2-DG. CellRox Green is a weak fluorescent dye that emits a stable fluorescent signal upon interaction with ROS. As visible in **Figure 3D**, 2-DG treated cells showed a significant increase of 30% of fluorescent signal, confirmed through a Welch´s two-tailed t-test, indicating that 2-DG can induce oxidative stress. Although NADPH depletion can induce ROS (10), oxidative stress was detectable at an earlier time point than the change in total NADP+/NADPH levels. This may be due to the fact that our NADP+/NADPH assay detects the overall decrease in NADP+/NADPH levels, but not the change in the ratio of NADP+ to NADPH, which might occur already at an earlier time point and triggers oxidative stress.

### 2.4 The NAD(P)H:quinone oxidoreductase is efficiently inhibited by 25nM ES-936

**Figure 4:**
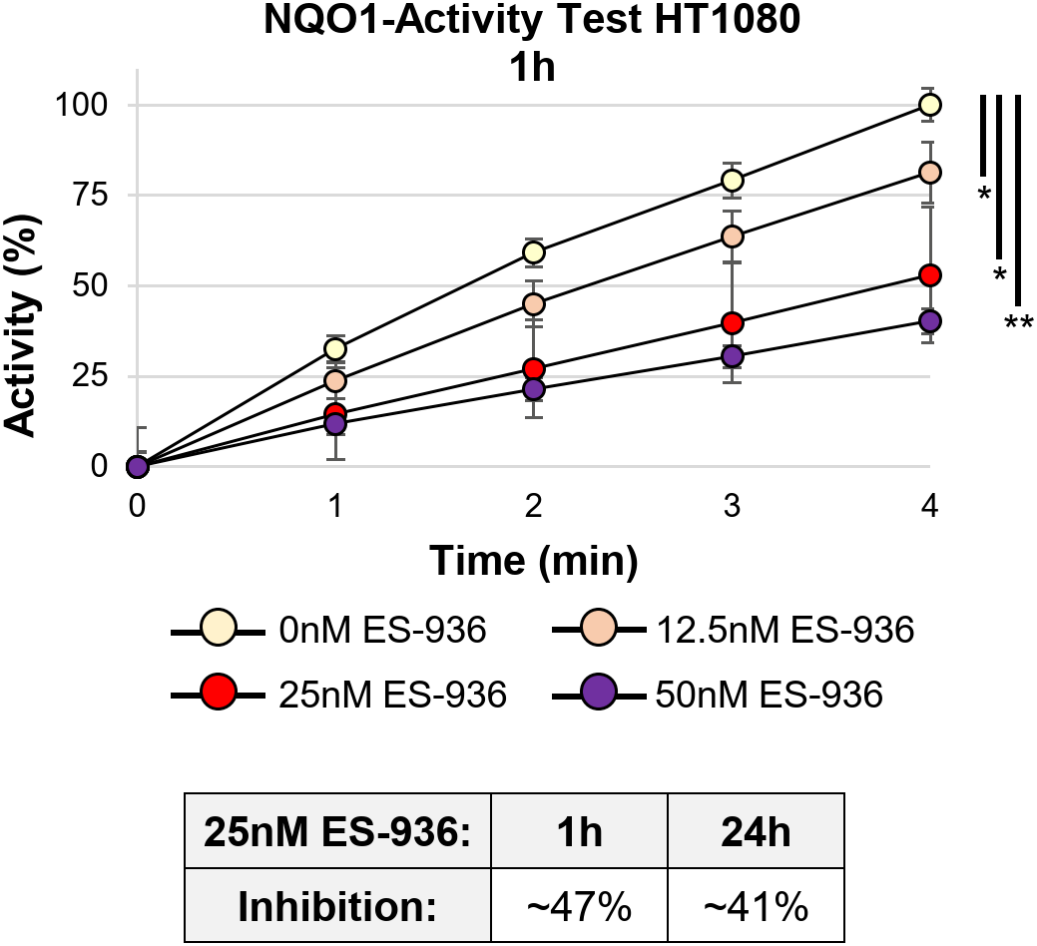
The NAD(P)H:quinone oxidoreductase (NQO1) is efficiently inhibited by 25nM ES-936. NQO1 activity in dependency of different concentrations of the inhibitor ES-936 after 1h treatment of HT1080 cells. Significance of NQO1 inhibition is shown at time point 4min. Lower Panel: Quantification of relative NQO1 inhibition at 25nM ES-936 after treatment for 1h or 24h indicating a long lasting response (N=1 for each 1h and 24h time point; n=2 techn. replicates; error bars indicate SEM). Significance was assessed using a Welch´s two-tailed t-test (*p<0.05, **p<0.001).

In the next step, we determined the efficiency of the NQO1 inhibitor 5-Methoxy-1,2-dimethyl-3-[(4-nitrophenoxy)methyl]indole-4,7-dione (ES-936). While no data of clinical studies with ES-936 are available, pre-clinical *in vitro* and *in vivo* analyses show that ES-936 induces a growth defect in human cancer cell lines and lead to a significant decrease in growth rates in xenograft tumors (43, 44). As the authors in these studies showed that 50nM or higher concentrations of ES-936 led already to a strong reduction of plating efficiency and anchorage-dependent growth (43), we decided to test lower ES-936 concentrations. For our experiment, HT1080 cells were treated with different concentrations of ES-936 for 1h and 24h, respectively. Subsequently, cell lysates were prepared and analyzed in an NQO1-activity test (**Supplementary Figure 2A**, **Figure 4**). As visible in **Figure 4**, 12.5nM ES-936 lead to an app. 19% inhibition of NQO1, while 25nM and 50nM ES-936 induced app. 47% and 60% inhibition of NQO1, respectively. The inhibitor-induced decrease of NQO1-activity was significant for all ES-936 concentrations tested at time point 4min (Welch´s two-tailed t-test; **Figure 4**). We decided to use 25nM ES-936 in our subsequent experiments as it exhibited a substantial and enduring inhibition of NQO1 of app. 41% after 24h of treatment (**Figure 4**, lower panel). In addition, this concentration also permitted the analysis of the inhibitor in combination with 2.5mM 2-DG and irradiation without lethal toxicity, as a 24h treatment of HT1080 cells with 25nM ES-936 alone did not induce a defect in cellular proliferation or a decrease in S-phase cell population (**Supplementary Figures 2B, C**). Using 25nM ES-936, we could not detect a change in the total NADP+/NADPH level in HT1080 cells after 4h and only a small decrease of app. 12% after 24h treatment (**Figure 3B, C**). Nevertheless, it was shown before that NQO1 affects the NAD(P)+/NAD(P)H ratio rather than the total NAD(P)+/NAD(P)H level (21). Additionally, we could not observe an increase in oxidative stress after 4h treatment with the rather low concentration of 25nM ES-936 alone (**Figure 3D**). Oxidative stress induced by ES-936 might be either persistent but below the detection limit of CellRox Green or requires a longer incubation time than 4h to be observable. In summary, 25nM ES-936 is an efficient inhibitor of the NQO1 enzyme and mildly affects NADP+/NADPH levels after 24h treatment at this concentration.

### 2.5 The combination of 2.5mM 2-DG and 25nM ES-936 acts additive on cell proliferation and affects NADP+/NADPH levels and ROS

**Figure 5:**
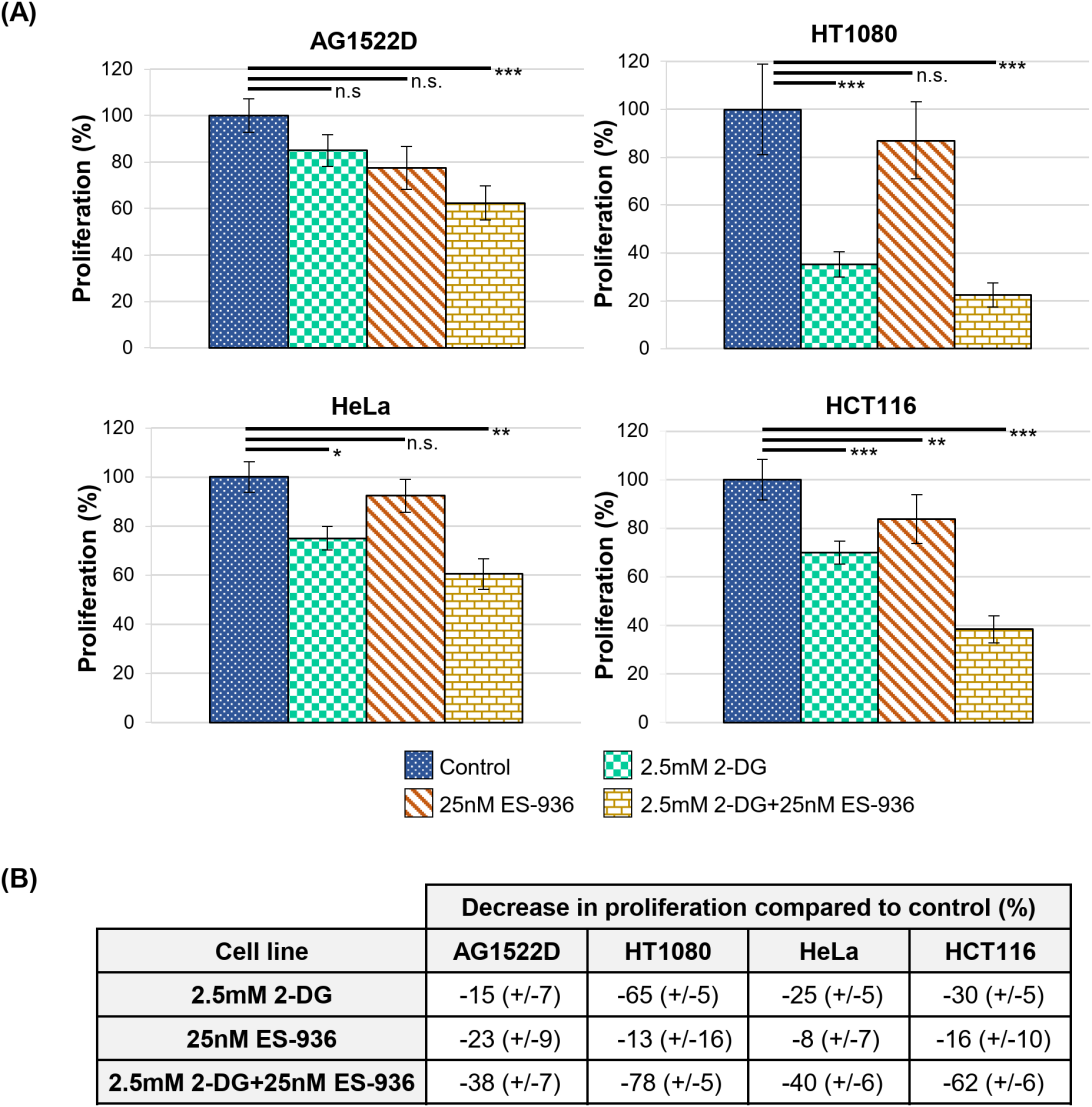
The combination of 2.5mM 2-DG and 25nM ES-936 acts additive on cell proliferation. **(A)** Relative cell proliferation of the indicated cell lines untreated or treated for 24h with 2.5mM 2-DG, 25nM ES-936 or the combination of both inhibitors. Subsequently, the inhibitors were removed and cells were grown for app. 10 days until the control samples reached 80% confluence. The protein mass was determined using a Sulforhodamin B assay as a surrogate measure of cellular proliferation. (N=4; n=8 techn. replicates; error bars indicate SEM). Significance was assessed using a Welch´s two-tailed t-test (n.s.= not significant, *p<0.05, **p<0.01; ***p<0.001). **(B)** Relative decrease in proliferation compared to controls (data from A).

Following the characterization of the single inhibitors 2-DG and ES-936, we incubated our cells lines for 24h in control medium or medium containing a combination of both 2.5mM 2-DG and 25nM ES-936 and subsequently cultivated the cells for app. 10 days in inhibitor-free medium to assess proliferation. When untreated control cells reached app. 80% confluence, we fixed and stained all samples with Sulforhodamin B to determine their protein content, which correlates to cell number and thus, proliferation. As visible in **Figure 5**, the treatment of the indicated cell lines with a combination of 2-DG and ES-936 led to a pronounced decrease of cell proliferation of about 38-78%, thus showing a mainly additive effect when compared to the effects of the single inhibitors. The normal fibroblast cell line AG1522D reacted least sensitive to 2-DG and the combination of 2-DG and ES-936, confirming our hypothesis that normal fibroblast cells can compensate 2-DG treatment better than cancer cells. In contrast, the normal fibroblast cells reacted similar to the inhibitor ES-936 than the cancer cell lines (**Figures 5A, B**), in line with the assumption that this inhibitor targets both normal fibroblast- and cancer cells. The decrease in proliferation was significant for the 2.5mM 2-DG and 2.5mM 2-DG+25nM ES-936 treated cancer cells lines, respectively (Welch´s two-tailed t-test). Although a slight proliferation defect of 25nM ES-936 treatment was visible in all cell lines, it was not significant except for the HCT116 cancer cells. The AG1522D fibroblasts showed only a significant decrease when treated with both, 2-DG and ES-936, although the observed proliferation defect was weaker than in the tested cancer cell lines (**Figures 5A, B**). Additionally, the effect of the inhibitor combination was tested in regard to NADP+/NADPH levels and ROS. For this, HT1080 cells were treated with the inhibitors and assessed after 4h and 24h, respectively. While a 4h incubation with both inhibitors did not show any change in NADP+/NADPH level, it was significantly decreased after 24h incubation, being only slightly lower than the level of 2-DG treated cells (**Figure 3B, C**). This is in line with the observation that a combination of 2-DG and ES-936 led to a pronounced reduction of the mitochondrial amount of the reduced forms of NAD(P)H in HT1080 cells after 6h incubation time (**Supplementary Figure 1E**). For this experiment, 2-Photon Fluorescence Lifetime Imaging Microscopy (2P-FLIM) was used to image and quantify mitochondrial NAD(P)H lifetime decay (45). An increase of oxidative stress in HT1080 cells was again detectable 4h after treatment with both inhibitors similar to that of 2-DG treated cells (**Figure 3D**). In summary, the combination of 2-DG and ES-936 showed a combined, robust inhibitory effect on cell proliferation under stress-free conditions in our tested cancer cell lines, while normal fibroblast AG1522D cells were also affected but to a lesser extend when compared to the cancer cell lines (**Figure 5**). The decrease of the NADP+/NADPH level and increase in ROS in HT1080 cells was similar to 2-DG treated cells after 24h treatment.

### 2.6 2.5mM 2-DG and 25nM ES-936 treated cancer- but not normal cells are radio-sensitized to low- and high-LET irradiation

**Figure 6:**
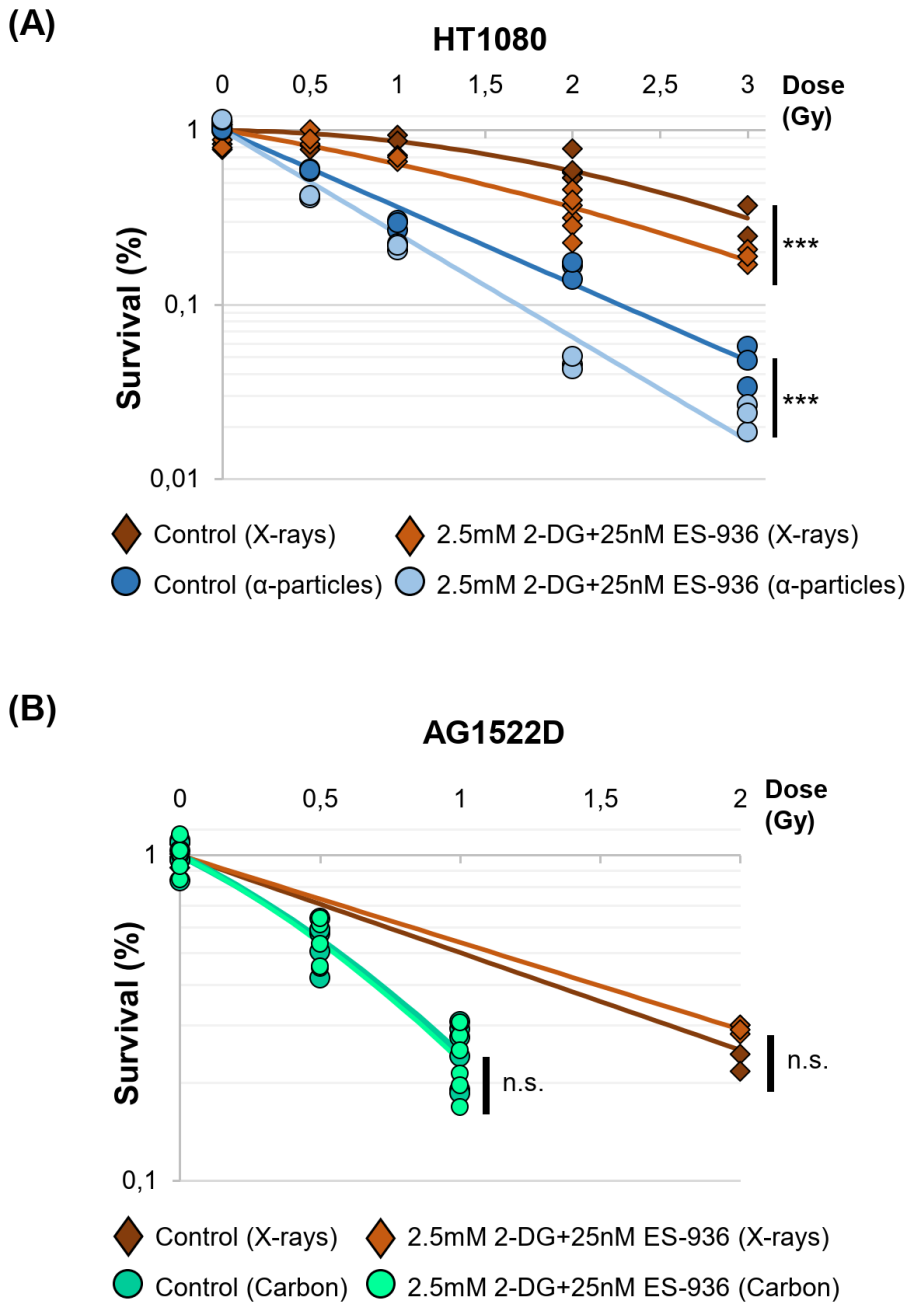
2.5mM 2-DG and 25nM ES-936 treated cancer- but not normal cells are radio-sensitized to low- and high LET-irradiation. **(A)** Clonogenic survival of HT1080 cancer cells and **(B)** normal AG1522D fibroblasts untreated or treated for 4h with 2.5mM 2-DG and 25nM ES-936 and subsequently irradiated with low-LET X-rays, or high-LET α-particles or Carbon-ions. 24h after irradiation, cells were seeded in inhibitor-free medium and analyzed after app. 10 days when colonies of more than 50 cells had formed. Significance of the survival curves are shown. (HT1080 X-rays: N=2 for dose points 0Gy and 2Gy; N=1 for dose points 0.5Gy, 1Gy and 3Gy, n=3 techn. replicates; HT1080 alpha-particles: N=1, n=3 techn. replicates; AG1522D X-rays: N=1, n=3 techn. replicates; AG1522D Carbon: N=2, n=3 techn. replicates). Significance was assessed: for 2 dose points: Welch´s two-tailed t-test; 3–5 dose points: Chi-squared method (46); ***p<0.001; n.s.= not significant).

To test whether inhibitor-treated normal fibroblast- and cancer cells react differently when challenged with radiation, we incubated normal AG1522D fibroblasts and HT1080, HeLa and HCT116 cancer cells with a combination of 2.5mM 2-DG and 25nM ES-396 for 4h. Subsequently, cells were irradiated with low- or high LET radiation and seeded for colony formation in inhibitor-free medium 24h post irradiation. In this experimental setting, DNA-repair takes place in the presence of the inhibitors, while colony formation proceeded in inhibitor-free medium. As shown in **Figure 6A**, the cancer cell line HT1080 was sensitized to both, low- and high LET irradiation (X-rays and alpha-particles, respectively) when treated with both inhibitors, visible through decreased colony formation. The cancer cell lines HeLa and HCT116 also showed – to a different extend – radiosensitization after treatment with both inhibitors and low- or high LET irradiation (**Supplementary Figures 3A, B**). In contrast, the normal fibroblast cell line AG1522D was not sensitized to low- and high LET irradiation (X-rays and Carbon-ions, respectively) when treated with a combination of both inhibitors (**Figure 6B**). Significance was assessed as indicated with either a Welch´s two-tailed t-test or the Chi-squared method (46). In summary, the combination of 2-DG and ES-936 seems to negatively affect the colony formation ability of different cancer cell lines after irradiation with low- or high LET but not of the normal fibroblast cell line AG1522D.

### 2.7 2-DG and ES-936 treated cancer- but not normal cells exhibit a DNA repair defect after high-LET irradiation that can be rescued by GTP

**Figure 7:**
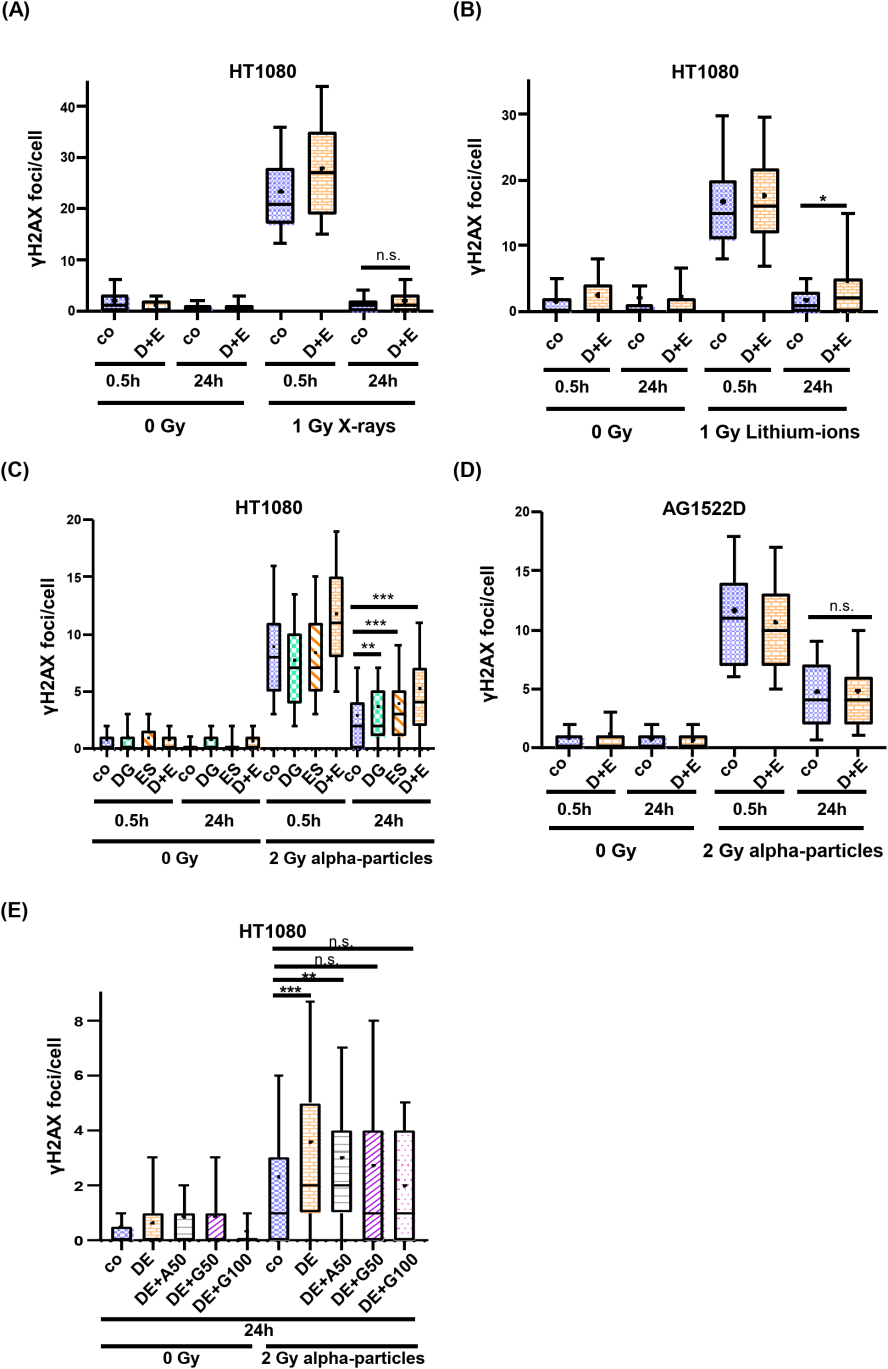
2-DG and ES-936 treated cancer- but not normal cells exhibit a DNA repair defect after high LET-irradiation. Evaluation of yH2AX foci as marker of DSB repair in HT1080 **(A–C)** and AG1522D **(D)** cells. Cells were either untreated or treated for 4h with the indicated inhibitors and subsequently irradiated with 1Gy X-rays and Lithium-ions, respectively, or 2Gy alpha-particles. Number of yH2AX foci per cell nuclei were analyzed for at least 100 nuclei per indicated time points and conditions. [**(A)** N=2; **(B)** N=1; **(C)** N=4 for co and DE and N=2 for DG and ES; **(D)** N=2; Box plots: whiskers=10-90%, += mean, - = median; co=control; D+E=2.5mM 2-DG+25nM ES-936; DG=2.5mM 2-DG; ES=25nM ES-936]. Significance was assessed using a Kolmogorov-Smirnov test (n.s.= not significant, *p<0.05, **p<0.01; ***p<0.001). **(E)** HT1080 cells were either untreated or treated with 50µM Adenosine, 50µM- or 100µM Guanosine 24h prior irradiation. 4h prior irradiation, cells were either untreated or treated with 2.5mM 2-DG and 25nM ES-936. 24h post irradiation with 2Gy alpha-particles, cells were PFA fixed, antibody stained and analyzed with a confocal microscope. Number of γH2AX foci per cell nuclei were analyzed for at least 100 nuclei per indicated time points and conditions. (N=2 for co, DE, DE+G50; N=1 for DE+A50, DE+G100; n=1 techn. replicate; Box plots: whiskers=10-90%, += mean, - = median; co=control, DE=2.5mM 2-DG+25nM ES-936, A50: 50µM Adenosine, G50: 50µM Guanosine, G100: 100µM Guanosine). Significance was assessed using a Kolmogorov-Smirnov test (n.s.= not significant, **p<0.01; ***p<0.001).

In the next step, we examined the DNA repair efficiency of inhibitor-treated and irradiated cells. For this, cells were treated with 2.5mM 2-DG and 25nM ES-936 for 4h and subsequently irradiated with either low- or high-LET radiation. Cells remained in inhibitor-containing medium up to 24h post irradiation and were fixed and immunostained at the indicated time points. Double-strand break (DSB) repair was analyzed using yH2AX foci as a read-out at 0.5h post irradiation to assess induction of DNA DSBs and at 24h post irradiation to detect residual damage (**Supplementary Figure 4**). While the inhibitor-treated cancer cell line HT1080 did not exhibit a significant difference in residual yH2AX foci numbers to untreated cells 24h after irradiation with 1Gy or 2Gy of X-rays, a significant DNA repair defect could be observed after irradiation with 1Gy Lithium-ions or 2Gy alpha-particles (**Figures 7A–C**, **Supplementary Figure 5A**). As visible in **Figure 7C**, both single inhibitors contributed to the observed DNA repair defect in an additive manner in the HT1080 cancer cells. A DNA-repair defect was also observable in inhibitor-treated HeLa cells after irradiation with Lithium-ions and alpha-particles, respectively (**Supplementary Figures 5C, D**). Because S-phase cells are more susceptible to the formation of yH2AX foci due to ongoing replication that can convert base damages or DNA single-strand breaks into DSBs, we used EdU pulse labeling to identify S-phase cells and excluded them from our evaluation. As shown in **Supplementary Figure 5B**, S-phase cells did not contribute significantly to the observed DNA repair defect in HT1080 cells, confirming that the detected DSB-repair defect was based on irradiation induced DSBs. To exclude the possibility that the residual numbers of yH2AX foci in inhibitor-treated HT1080 cancer cells 24h post high-LET irradiation were based on apoptotic cells, we performed a TUNEL-assay. In **Supplementary Figure 6**, it becomes visible that a high-LET irradiated HT1080 population contains no detectable apoptotic cells 24h after irradiation compared to DNAseI- or Staurosporine-treated or low-LET (X-ray) irradiated cells. Consequently, the elevated number of detected yH2AX foci seem not to be based on apoptotic cells. As a comparison, we analyzed whether normal AG1522D fibroblasts also showed a DNA-repair defect after inhibitor treatment and high-LET irradiation. In contrast to the cancer cell lines tested, no repair defect could be detected in the AG1522D cells 24h after alpha-particle irradiation (**Figure 7D**). We conclude that the inhibitor combination of 2-DG and ES-936 induces a DNA-repair defect after high- but not low-LET irradiation in our tested cancer cell lines but not in normal fibroblast AG1522D cells. Significance was assessed using a Kolmogorov-Smirnov test. Finally, we tested the hypothesis of Zhou et al. (47) that connects metabolism and DNA repair with each other. The authors establish in their work that specifically GTP activates a signaling cascade that promotes non-homologous end joining (NHEJ). To examine whether 2-DG and ES-936 can affect irradiation induced DSB repair through interference with GTP availability, HT1080 cells were supplemented 24h prior irradiation with either 50µM Adenosine or 50µM and 100µM Guanosine, respectively. Adenosine and Guanosine can enter the cells where the molecules are converted to ATP and GTP, respectively (47). 4h prior irradiation with 2Gy alpha-particles, cells were treated with 2.5mM 2-DG and 25nM ES-936. DNA repair efficiency was assessed again 24h post irradiation. As visible in **Figure 7E**, the addition of Guanosine but not Adenosine decreased the number of DNA damage foci after 24h significantly (Kolmogorov-Smirnov test), implying that specifically GTP can mediate DSB repair and that the inhibitor combination of 2-DG and ES-936 could indeed interfere with GTP availability.

## 3 Discussion

The objective of this study was to sensitize cancer cells to high-LET irradiation (high-linear energy transfer) in order to enhance the efficacy of future cancer therapies. The present study demonstrates that the HT1080, HeLa and HCT116 cancer cell lines exhibit increased sensitivity to both low- and high-LET irradiation when treated with the inhibitors 2-DG and ES-936, while normal AG1522D fibroblasts are not radio-sensitized. The inhibitors decrease clonogenic survival after low- and high-LET irradiation and interfere with DNA repair after high-LET irradiation via distinct mechanisms. The findings of this study offer a valuable contribution to the field by providing a more comprehensive understanding of the effects of 2-DG and ES-936 at different radiation qualities. This enhanced knowledge may also facilitate the optimization of their utilization in relation to the quality of irradiation and potential therapeutic outcomes.

Previous studies already targeted the cancer cell specific metabolism, which employs glycolysis to generate energy and metabolites to allow faster cellular growth, to sensitize cancer cells to irradiation (14, 32, 36). During our analyses, we discovered that our tested normal fibroblast- and cancer cell lines did not exhibit a clear preference for oxidative phosphorylation and aerobic glycolysis, respectively as suggested in the literature (4) (**Figure 1B**). We assume that in contrast to fresh tumor samples, our cell lines might have already adjusted their metabolism to the artificial cell culture environment. Nevertheless, the normal fibroblast cell lines exhibited a large respiratory spare capacity under metabolic stress conditions, while the cancer cell lines possessed either a large glycolytic spare capacity or no spare capacities at all (**Figure 2C**). Consequently, the cancer cell lines were still targeted by the inhibitor 2-DG, which can interfere with active glycolysis and the glycolytic spare capacity. We could show that 2-DG did not only lead to a decrease of glycolytic ATP production, it also induced a G1/S cell cycle arrest, a proliferation defect and a decrease in NADP+/NADPH levels and increase in ROS (**Figure 3** and **Supplementary Figure 1**). It was shown before that 2-DG also affects the glycolysis-linked PPP (9, 12), which produces pre-cursors for nucleotides and NADPH that can be used for the redox defense. Consequently, a 2-DG induced depletion of nucleotides could explain our observed G1/S cell cycle and proliferation defect and NADPH-dependent increase in ROS. Although we could only detect a decrease of total NADP+/NADPH levels after 24h 2-DG treatment but an increase of ROS already after 4h treatment, we assume that both results are connected. With our assay, we can detect a change in total NADP+/NADPH levels but not of the redox-ratio. We assume that 2-DG treatment might already decreases NADPH at an earlier time point and consequently changes the NAPD+/NADPH ratio but that a total decrease of NADP+/NADPH levels is detectable only at a later time point. To further target the redox-defense, we also treated our cell lines with the NQO1-inhibitor ES-936 to interfere with the NAD(P)+/NAD(P)H balance and increase ROS (20, 21). ES-936 is an inhibitor that is currently tested pre-clinical *in vitro* and *in vivo* experiments but not yet in clinical studies (43, 44). Although its applicability in humans has to be still assessed, *in vitro* and vivo studies showed that it induces a growth defect in human cancer cell lines, leads to a significant decrease in growth rates in xenograft tumors and shows a potent inhibition of NQO1 in most tissues of mice (43, 44, 48). In addition, the authors showed that ES-936 exhibited a biostability over hours and could induce DSB-breaks, especially at doses higher than 100 nM (43). Although its uptake and inhibitory and potentially off-target effects of NQO1 in humans still has to be tested to confirm its suitability for cancer therapy, its *in vitro* and *in vivo* properties, even including its potential to induce DSBs, are promising to be used to radiosensitize cancer cells and to hamper DSB-repair. This inhibitor targets both, normal fibroblast- and cancer cells. However, cancer cells were expected to react more sensitive due to their faster metabolism that can also induce higher base levels of ROS and a higher dependence on NQO1 to counteract oxidative stress, respectively. With this approach, we aimed to increase the oxidative stress level, which should interfere with irradiation-induced DNA damage and subsequent DSB repair. Although we could only detect a slight decrease of NADP+/NADPH levels after 24h of ES-936 treatment and no increase in ROS after 4h treatment in HT1080 cancer cells, we assume that ES-936 induces either a chronic oxidative stress level below detection of our assays or a slow accumulation of oxidative stress, which is not detectable within 24h treatment (**Figure 3**). These assumptions are based on the results that a 24h incubation of cells with ES-936 followed by a 7–10 day incubation with inhibitor-free medium induced a proliferation defect in normal- as well as cancer cells that was additive when cells were additionally treated with 2-DG (**Figure 5**). This is in line with the observation that a combination of 2-DG and ES-936 lead to a pronounced reduction in the reduced forms of NAD(P)H in HT1080 cells already after 6h incubation time (**Supplementary Figure 1E**). Even normal AG1522D fibroblasts showed a certain degree of proliferation reduction, implying that the irreversible inhibition of NQO1 by ES-936 induces either oxidative stress or interferes with NAD(P)+/NAD(P)H dependent processes. Nevertheless, the cancer cells reacted more sensitive to the single inhibitors and their combination, respectively, than the normal fibroblast cells. While normal fibroblasts are not strongly affected by 2-DG due to their different and slower mechanism and consequently should compensate oxidative stress better, we hypothesized that 2-DG and ES-936 treated cancer cells will have problems to handle DNA repair in context of lack of energy and increased oxidative stress. When we treated our cells lines with both inhibitors and irradiated them with either low- or high-LET radiation, the cancer cells exhibited a decreased colony formation ability in contrast to normal fibroblast AG1522D cells (**Figure 6** and **Supplementary Figure 3**). Under challenging conditions, as it is the case for irradiated cells, the hampered metabolism and oxidative stress might drive cancer cells to their limit to sustain their fast cell growth and to repair irradiation induced DNA damage, whereas the normal fibroblast cells are less affected. The latter ones possess a slower proliferation rate and therefore probably a lower demand of energy and metabolites as well as less oxidative stress. We also showed that cancer- but not normal fibroblast cells treated with 2-DG and ES-936 exhibited a DNA repair defect, which became significant solely after high-LET irradiation (**Figure 7**; **Supplementary Figure 5**). Further analysis showed that both inhibitors contributed to this defect and acted in an additive manner. Critical for this phenotype seems to be a longer incubation time (at least 2h) of cells with the inhibitors prior to irradiation. The observed DNA repair defect was not associated with either S-phase cells or apoptosis. (**Supplementary Figures 5,6**). Previously, it was shown that 2-DG hampers the PPP, which also provides ribose 5-phosphate as a source for nucleotide synthesis (9; 42). Because our experiments showed a 2-DG induced G1/S checkpoint arrest and a decreased S-phase cell population, we speculate that these phenotypes could origin from a depleted nucleotide pool, which might also affect DNA repair. A reduction of NAD(P)H pool as observed after combined inhibitory conditions (**Supplementary Figure 1E**) can lead to disruptions in various cellular processes. Besides being essential for maintaining the cellular redox balance and combating oxidative stress, NADPH is crucial for anabolic reactions by providing reducing equivalents for synthesizing essential molecules like fatty acids, steroids, and some amino acids. These are not only necessary for protein synthesis, but also for other molecules including nucleotides. As NAD(P)H is also essential for the reduction of ribonucleotides to deoxyribonucleotides by ribonucleotide reductase (49), we hypothesize that a depletion of this reducing equivalent might act additive or synergistic to a nucleotide pool perturbation after 2-DG treatment. Interestingly, during our studies, data from 47 were published, indicating that the nucleotide GTP can activate a signaling cascade that promotes NHEJ by the activation of Rac1/Abi-1. To test the hypothesis that GTP depletion could hamper DSB-repair in our inhibitor treated and high-LET irradiated cancer cells, we supplemented HT1080 cells with either guanosine or, as a negative control, adenosine. Once within a cell, both nucleosides are converted to GTP and ATP, respectively. The observation that the DNA-repair defect was only visible after at least 2h incubation time of cells with inhibitors prior irradiation might be explained by the time required to disturb the balance of nucleotide synthesis and to lead to a depletion of GTP that apparently can also function as signaling molecule (47). We could show that supplementation with guanosine but not adenosine rescued the observed DNA repair (**Figure 7E**), implying that indeed GTP might exert a function in the promotion of DSB-repair. The observed rescue also argues against a generally depleted nucleotide pool being responsible for the repair defect. However, we have to state that further experiments like measuring Rac1/Abi-1 activation and quantification of the nucleotides would be necessary to pinpoint the exact molecular mechanisms of the inhibitor´s interference with repair regulation. In addition, measuring global DSB-repair using yH2AX assay cannot provide direct evidence for the affected repair pathway. Further studies might utilize NHEJ-specific markers or assays in order to clarify the role of GTP-dependent regulation for this pathway upon 2-DG treatment. For the normal fibroblast AG1522D cell line, no DNA repair defect was observed after inhibitor-treatment and high-LET irradiation. We assume that normal fibroblasts have again an advantage due to their slower metabolism and cellular proliferation. Their nucleotide pool might not be as fast exhausted or misbalanced, as it is the case for the cancer cells, allowing them to use GTP to mediate DNA repair. Implementing additional normal fibroblast cell lines in follow-up experiments could strengthen these finding of a differential response of normal fibroblasts in comparison of cancer cells. Initial experiments with a second fibroblast cell line (NFFp) already showed similar metabolic properties to AG1522D cells. These properties include large oxidative and small glycolytic spare capacities, which imply a similar response to the tested inhibitors to that observed in AG1522D cells. However, this requires validation in future experiments (**Figures 1**, **2** and **Supplementary Figure 1**). As shown before, high-LET irradiation induces more clustered DNA damage than low-LET irradiation (28–30). The high-LET radiation induced complex DNA damage is frequently processed by resection dependent repair pathways (50). DSB repair by end joining (EJ) can subdivide in several, error-prone and also resection-dependent pathways that might be activated through GTP dependent signaling. Dealing with complex DNA damage is more challenging, especially for the fast growing cancer cells and could consequently deplete required resources like e.g. GTPs faster than the repair of low-LET induced DNA damage, thus providing a basis for the observed LET dependency. Further studies will have to investigate this mechanism and the dependencies on specific repair pathways in more detail to enhance the observed effects of the inhibitors for irradiation therapy and especially high-LET irradiation therapy.

## 4 Materials and methods

### 4.1 Tissue culture

The cancer cell lines HT1080 (CLS), HeLa (ATCC) and HCT116 (kindly provided by B. Vogelstein) were cultured in DMEM with either 4.5 g/L (high) (Gibco) or 1.0 g/L (low) (Sigma-Aldrich) glucose, 10% FCS, 1% Glutamine and 1% Penicillin/Streptomycin at 37°C and 95% humidity. The normal fibroblast cell lines AG1522D (Coriell) and NFFp (51) were cultured in DMEM with either high- or low glucose, 20% FCS, 1% Glutamine, 1% non-essential amino acids and 1% Penicillin/Streptomycin at 37°C and 95% humidity. Cell lines were maintained in high glucose medium, while experiments were performed in low glucose medium to allow a 1:2 ratio of 2-DG and glucose.

### 4.2 Cellular metabolism

A Seahorse XFp analyzer (Agilent) was used to characterize the metabolic properties of the indicated cell lines. Cells were analyzed 24h post seeding, following the manufacturer´s protocol for the Real-Time ATP Rate Assay, Cell Mito Stress Test and Glycolysis Stress Test.

### 4.3 Proliferation assay

Cells were seeded to obtain 30% confluence and 24h later treated with 2.5mM 2-DG in low glucose medium to allow a 1:2 ratio of 2-DG and glucose. After a 24h incubation time, untreated and treated cells were counted using a coulter counter (Beckman). Untreated samples were set as 100% proliferation and treated samples were normalized to this value.

### 4.4 Cell cycle distribution

Cells were seeded to obtain 30% confluence and 24h later treated with 2.5mM 2-DG in low glucose medium to allow a 1:2 ratio of 2-DG and glucose. After a 24h incubation time, cells were harvested, fixed with ice cold 70% EtOH and stained with DAPI. Cell cycle distribution and amount of dead cells/debris were analyzed with a CytoflexS (Beckman Coulter) and FlowJo (BD) software.

### 4.5 NADP/NADPH assays

Cells were seeded into a 96-well plate to obtain 30% confluency and 24h later treated with the indicated inhibitors in low glucose medium. After 4h and 24h, cells were processed following the manufacturer´s protocol of the NADP/NADPH-Glo assay (Promega). For the 24h time point, cell numbers were normalized to account for the altered cell proliferation due to treatment with 2-DG. For determination of the mitochondrial amount of NAD(P)H, cells were seeded in 35mm glass bottom petri-dishes (Greiner) and were either untreated or treated with the inhibitors for 6h prior analyses. 2-Photon Fluorescence Lifetime Imaging Microscopy (2P-FLIM) was used to image and quantify mitochondrial NAD(P)H lifetime decay (45). The 2P-FLIM setup is based on an Olympus IX71 microscope equipped with a Tokai Hit climate chamber (37°C, 5%CO2), a DCS-120 confocal FLIM System (Becker&Hickl) and a mode locked titanium sapphire laser (Coherent Chameleon II) operated at 720nm and 80MHz. Images were collected by time correlated single photon counting through a water immersion 60X 1.2 NA lens. Emission was recorded by a non-descanned HPM-100–07 hybrid detector (Becker&Hickl) after reflection by a long pass dichroic mirror and passing a FSP-680 blocking filter, a T500SPXR beam splitter and a 458/64 band-pass filter (all Semrock). Decay curves of the 2P-FLIM Images were generated by a bi-exponential fit fixing the fast decay time for free NAD(P)H to 350ps (SPCimage software Becker and Hickl). The quality of the fits were checked by Chi-squared and the uniform distribution of residuals. Pixel intensities and relevant fitting parameters were exported and pixels corresponding to the mitochondria analyzed using in-house written software (Image D; https://davideilenstein.github.io/ImageD/index.html).

### 4.6 Oxidative stress

Cells were seeded to obtain 30% confluence and 24h later treated with the indicated inhibitors in low glucose medium. After 4h, CellRox green (Invitrogen) was added for 30 minutes to the cells. Subsequently, cells were PFA fixed and analyzed with a fluorescent microscope.

### 4.7 NQO1-activity assay

Cells were seeded to obtain 30% confluence and 24h later treated with the indicated concentrations of ES-936 in low glucose medium. After 1h and 24h cells were processed following the manufacturer´s protocol of the NQO1 Activity Assay (Abcam). Protein concentrations were determined using a Pierce BCA Protein Assay (Thermo Fisher Scientific) and Spectramax i3X (Molecular Devices) and 25µg of all lysates were loaded onto a 10% SDS-gel to confirm the use of equal amounts of lysates in the assay.

### 4.8 S-phase cells

Cells were seeded to obtain 30% confluence and 24h later treated with 25nM ES-936 in low glucose medium. After 24h, 10µM EdU was added for 30 minutes to the cells. Subsequently, cells were PFA fixed and cells were processed following the manufacturer´s protocol of the EdU Cell Proliferation Kit for Imaging (Baseclick), using a 594nm antibody (BCL-EdU 594-1, Base-Click; AF594-Picolyl-Azide, Jena Bioscience) and imaged with a fluorescent microscope.

### 4.9 Sulforhodamin B assay

Cells were seeded in 96-well plates and 24h later treated with the indicated inhibitors in low glucose medium. After 24h, the medium was replaced by inhibitor-free, low glucose medium and cells were cultured for several days until the untreated control sample of each cell line reached 80% confluency. Subsequently, cells were fixed with 10% TCA and stained with 0.057% Sulforhodamin B in 1% acetic acid. The dye was removed from the fixed cells by adding 10mM Tris Base pH 10.5 and measured with a Spectramax i3x (Molecular Devices) to correlate the intensity of the dye solution with cellular proliferation (52). Control samples were set as 100% proliferation and treated samples were normalized to this value.

### 4.10 Clonogenic survival assay

Cells were seeded to obtain 30% confluence and 24h later treated with the indicated inhibitors in low glucose medium. After 4h, cells were irradiated with the indicated doses and radiation qualities and 24h later seeded in inhibitor-free, low glucose medium for colony formation. Depending on the cell lines, cells were cultured for up to 10 days until colonies with more than 50 cells were formed. Subsequently, colonies were fixed and stained with 3x Methylenblue for 30 minutes and counted for analysis.

### 4.11 DNA repair efficiency

Cells were seeded to obtain 30% confluence and 24h later treated with the indicated inhibitors in low glucose medium. After 4h, cells were irradiated with the indicated qualities of irradiation and PFA fixed after either 0.5h or 24h. Prior fixation, 10µM EdU was added to label S-phase cells. Subsequently, cells were processed following the manufacturer´s protocol of the EdU Cell Proliferation Kit for Imaging (Baseclick), using a 594nm antibody (BCL-EdU 594-1, Base-Click; AF594-Picolyl-Azide, Jena Bioscience), followed by antibody staining for yH2AX (05-636, Millipore; Alexa Fluor 488 A-11017, Invitrogen) and DAPI. Samples were imaged as z-stacks using a Leica SPE confocal microscope equipped with a 63x and 100x lens, respectively. Maximum intensity projections of the fluorescence image stacks were analyzed using manual foci counting in Fiji.

### 4.12 *In Situ* cell death detection

Cells were seeded to obtain 30% confluence and 24h later treated with the indicated substances in low glucose medium. After 4h, inhibitor-treated cells were irradiated with the indicated qualities of irradiation and PFA fixed after 24h. Subsequently, cells were processed following the manufacturer´s protocol of the *In Situ* Cell Death Detection Kit (Roche) and analyzed with a fluorescent microscope.

### 4.13 Irradiation qualities

For X-ray irradiation, an X-ray machine (MXR 320/26, GE Sensing & Inspection Technologies. Ahrensburg, Germany) with a tube voltage of 250 kV and app. dose rate of 2.0 Gy/min was used. Alpha-particle irradiation was performed employing an 241Am-source. Lithium-ion irradiation (1 Gy) was performed at the linear accelerator UNILAC of the GSI Helmholzzentrum für Schwerionenforschung GmbH (10.9 MeV/u, 39.5 keV/μm). Carbon-ion irradiation took place at the Heavy-ion Synchrotron (SIS) of the GSI Helmholzzentrum für Schwerionenforschung GmbH or at the Marburger Ionstrahl-Therapiezentrum with samples positioned in the midst of a 40mm SOPB (mean dose LET of app. 65 keV/µm; dose 0.5-1Gy).

### 4.14 Inhibitors

2-Deoxy-D-Glucose (Sigma); ES-936 (Santa Cruz).

### 4.15 Statistical analysis

´N´ indicates number of independent experiments. Technical replicates are given by ´n´. Significance was assessed using the following tests as indicated: one-way ANOVA and Tukey *post-hoc* multiple comparisons, Welch´s two-tailed t-test, Chi-squared method (46), Kolmogorov-Smirnov test; (n.s.= not significant, *p<0.05, **p<0.01; ***p<0.001). Error bars indicate standard error of the mean (SEM).

## Data Availability

The original contributions presented in the study are included in the article/**Supplementary Material**. Further inquiries can be directed to the corresponding author.
