# Supplementary figures and images for: 2-Deoxy-D-Glucose and ES-936 sensitize cancer- but not normal cells to both low- and high LET irradiation
Source: Front Oncol. 2025 Aug 18;15:1633299. doi: 10.3389/fonc.2025.1633299 (PMC12399609; doi:10.3389/fonc.2025.1633299)

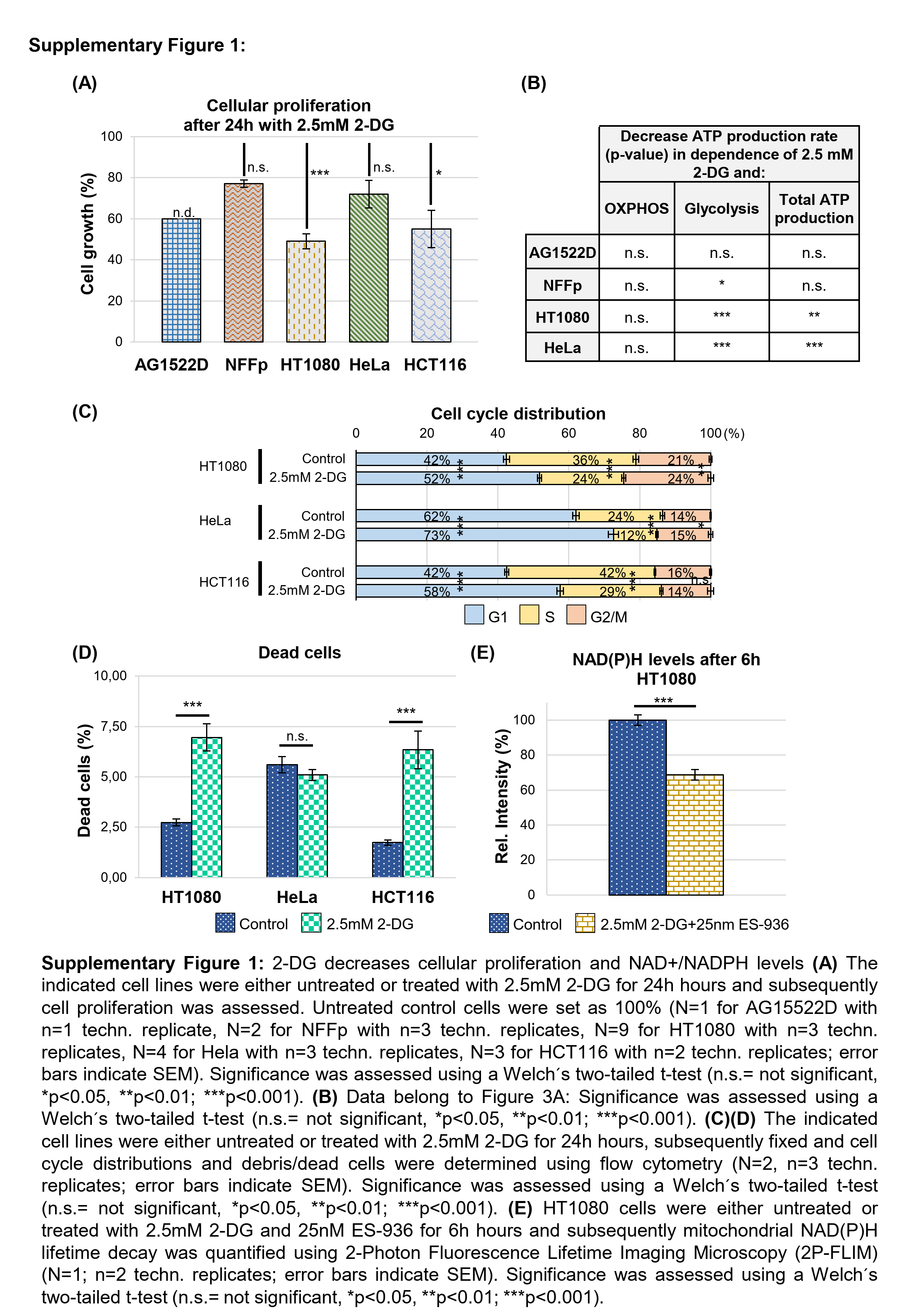

Supplement: Supplementary file 1 [file Image1.tif]

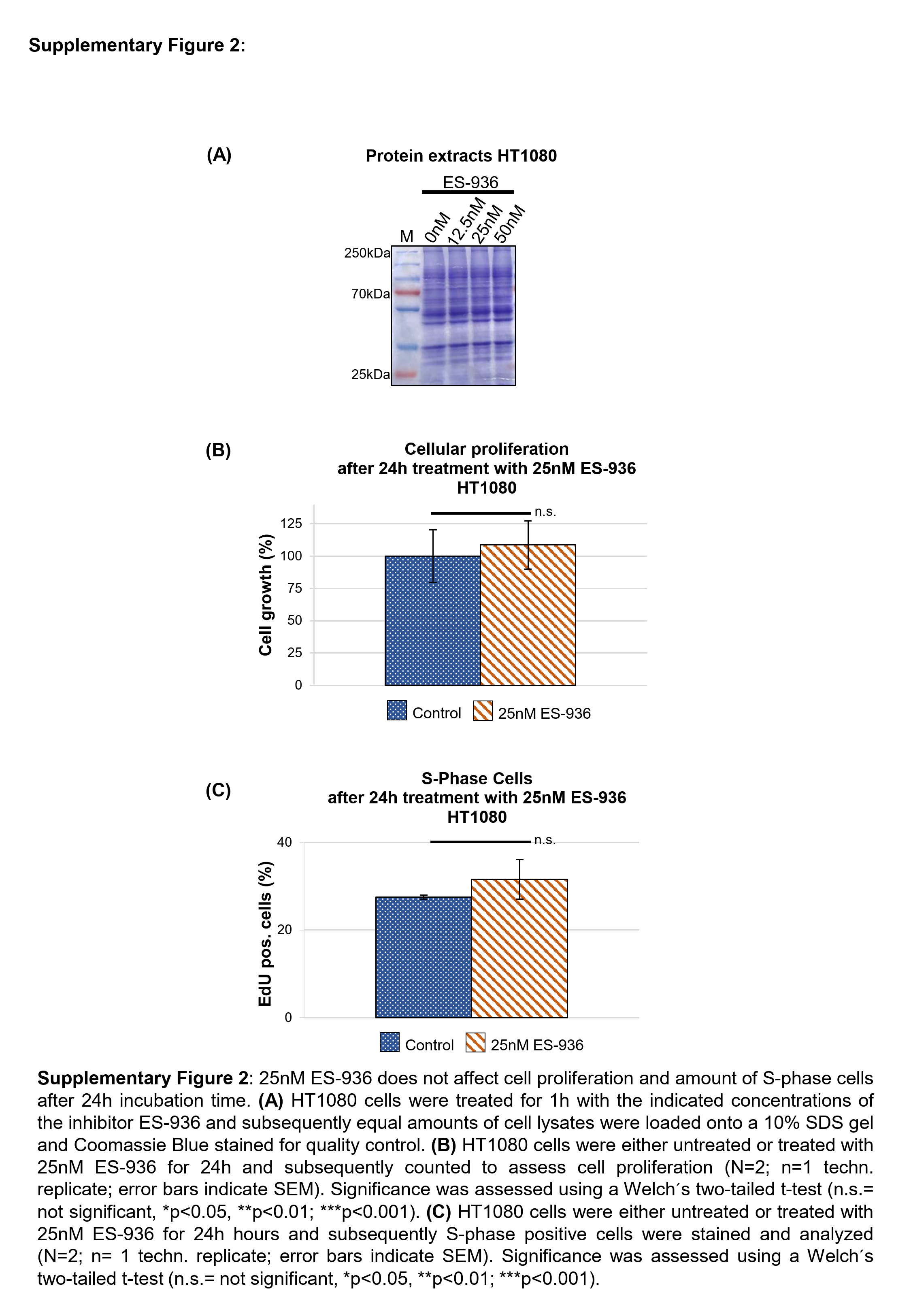

Supplement: Supplementary file 2 [file Image2.tif]

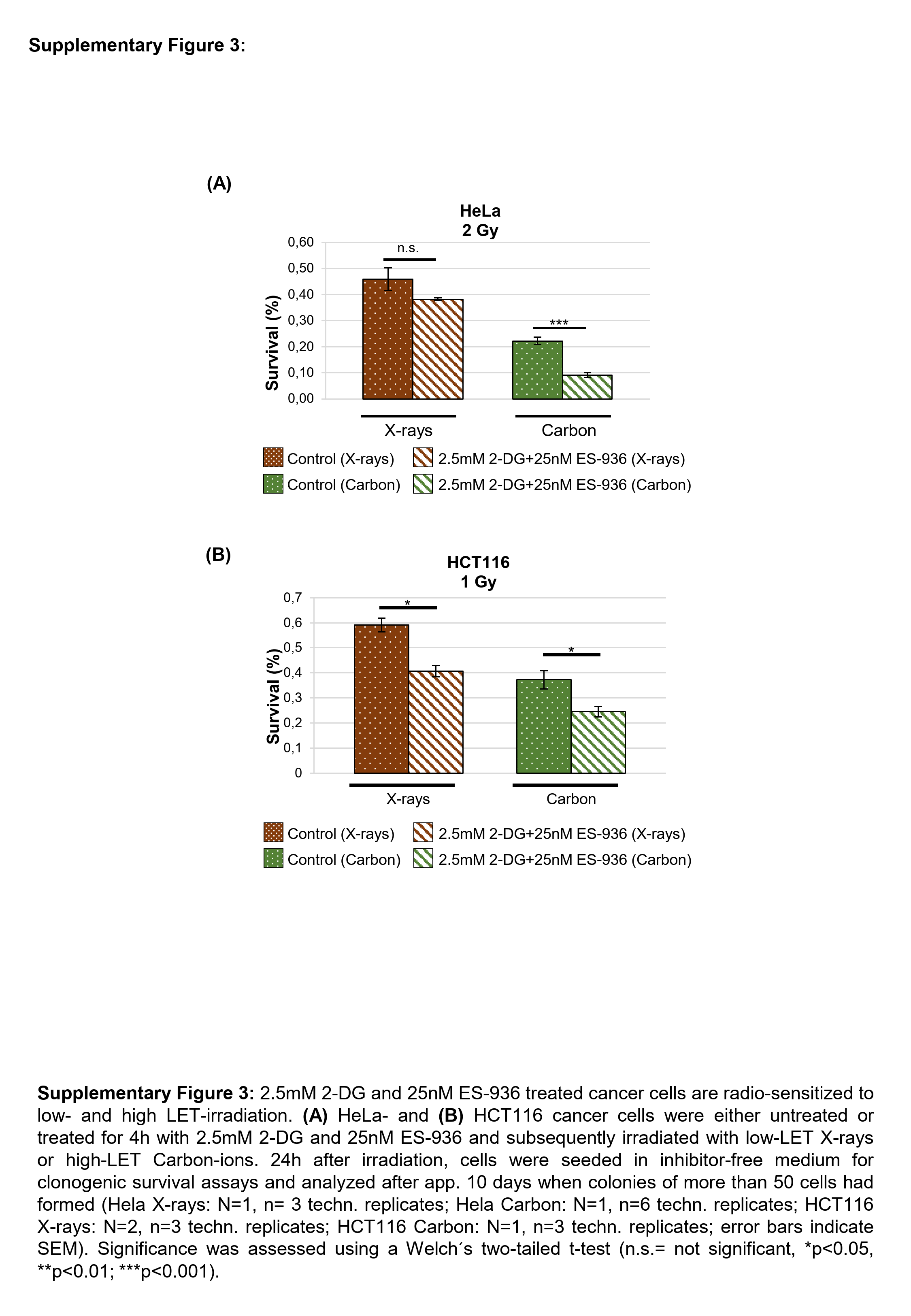

Supplement: Supplementary file 3 [file Image3.tif]

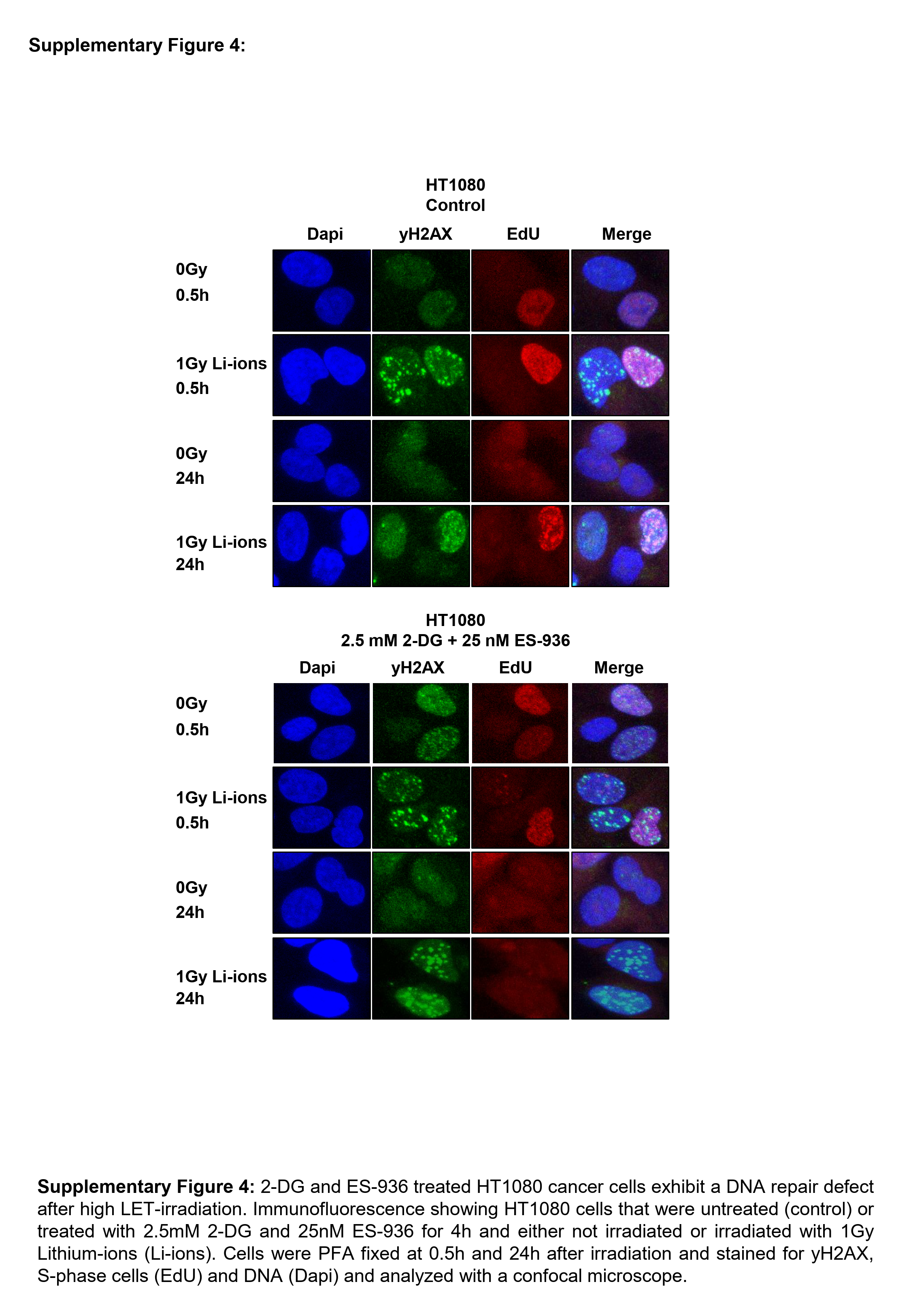

Supplement: Supplementary file 4 [file Image4.tif]

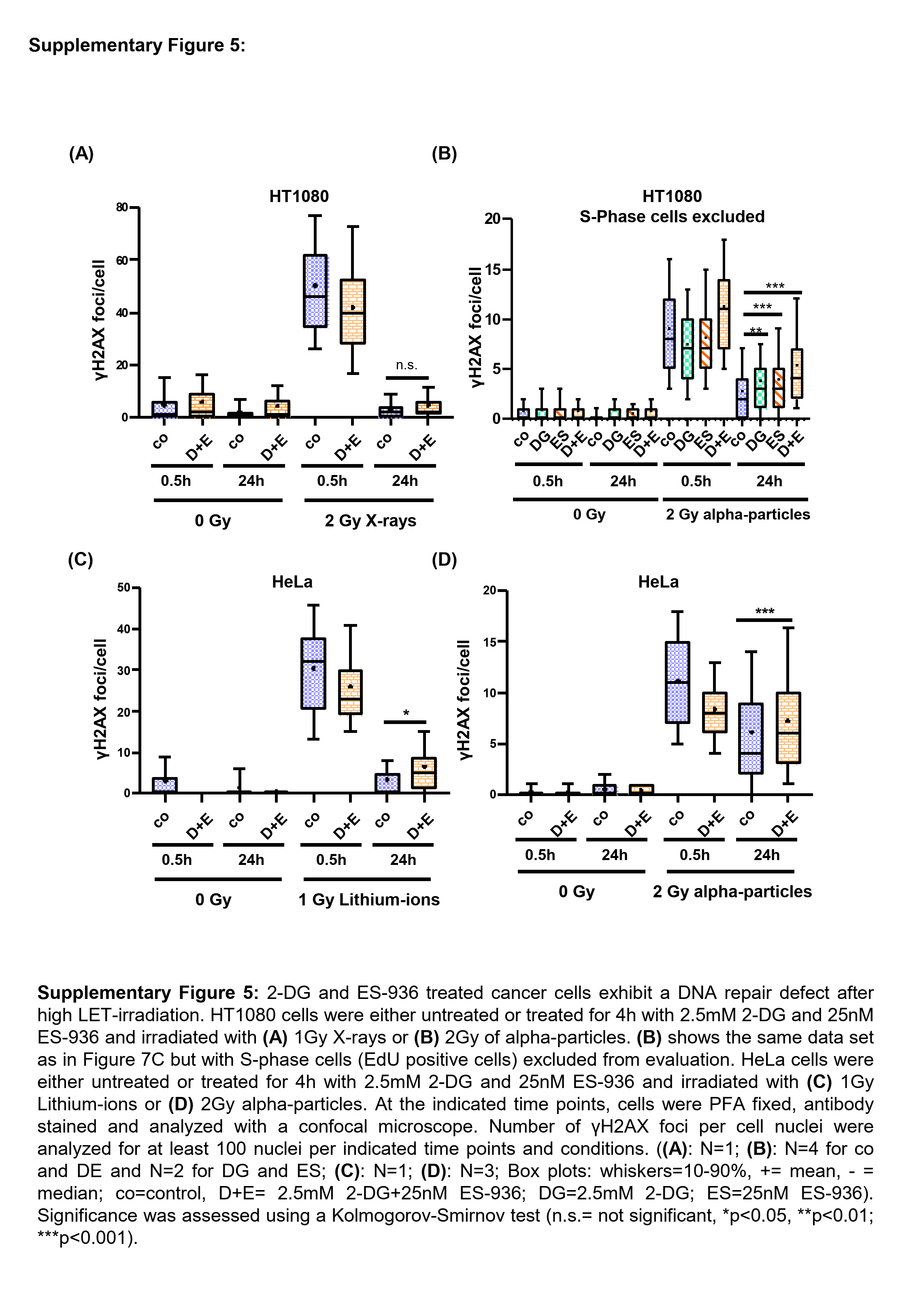

Supplement: Supplementary file 5 [file Image5.tif]

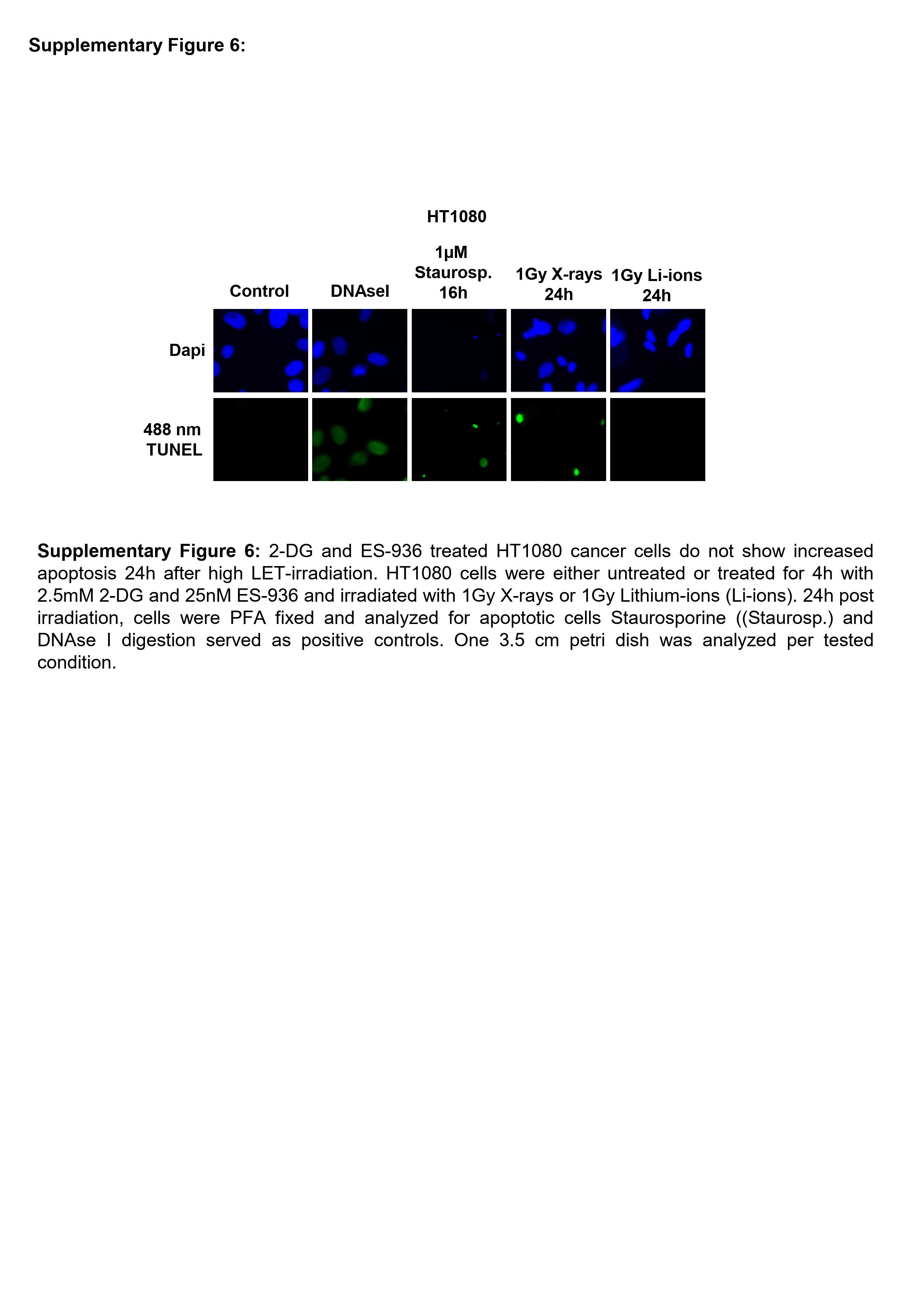

Supplement: Supplementary file 6 [file Image6.tif]
